# Analysis of adenoviral attachment to human platelets

**DOI:** 10.1186/1743-422X-6-25

**Published:** 2009-02-17

**Authors:** Nilly Shimony, Gregory Elkin, Dror Kolodkin-Gal, Lina Krasny, Simcha Urieli-Shoval, Yosef S Haviv

**Affiliations:** 1 Department of Medicine, Hadassah-Hebrew University Medical Center, Jerusalem, Israel 91120; 2 Department of Virology, Hadassah-Hebrew University Medical Center, Jerusalem, Israel 91120; 3 Department of Hematology Mount Scopus, Hadassah-Hebrew University Medical Center, Jerusalem, Israel 91120

## Abstract

**Background:**

Systemic adenoviral (Ad) vector administration is associated with thrombocytopenia. Recently, Ad interaction with mouse platelets emerged as a key player determining liver uptake and platelet clearance. However, whether Ad can activate platelets is controversial. Thus, *in vitro *analysis of Ad attachment to platelets is of interest.

**Methods:**

We developed a direct flow cytometry assay to specifically detect Ad particles adherent to human platelets. The method was pre-validated in nucleated cells. Blocking assays were employed to specifically inhibit Ad attachment to platelets. Platelet activation was analyzed using annexin v flow cytometry.

**Results:**

We found *in vitro *that Ad binding to human platelets is synergistically enhanced by the combination of platelet activation by thrombin and MnCl2 supplementation. Of note, Ad binding could activate human platelets. Platelets bound Ad displaying an RGD ligand in the fiber knob more efficiently than unmodified Ad. In contrast to a previous report, CAR expression was not detected on human platelets. Integrins appear to mediate Ad binding to platelets, at least partially. Finally, αIIbβ3-deficient platelets from a patient with Glanzmann thrombasthenia could bind Ad 5-fold more efficiently than normal platelets.

**Conclusion:**

The flow cytometry methodology developed herein allows the quantitative measurement of Ad attachment to platelets and may provide a useful *in vitro *approach to investigate Ad interaction with platelets.

## Background

Thrombocytopenia is a major adverse effect of high dose systemic administration of adenoviral (Ad) gene therapy vectors. While a previous report did not find platelet activation by Ad [1], recent studies have shown that Ad may activate platelets [2] and binds *in vivo *to murine thrombocytes resulting in hepatic sequestration [3]. Ad-induced thrombocytopenia has been shown to be dose-dependent, saturable and reversible [4], compatible with a ligand-receptor mechanism. Recently, binding of Ad to platelet was indirectly suggested following interference of platelet adhesion to fibronectin after incubation with Ad [2]. In this study we developed a direct flow cytometry assay to quantitatively analyze Ad attachment to human platelets *in vitro *and to characterize their interaction.

Many microorganisms in addition to Ad have evolved to facilitate cell entry via RGD recognition of cell surface integrins. For example, integrins mediate RGD-dependent attachment of picornaviruses [5,6] and bacteria [7,8]. In contrast, Group C Ad primarily attaches to the cell surface via the fiber protein knob binding to CAR [9] (coxsackie and Ad receptor). Next, Ad internalizes primarily utilizing αVβ3 integrin [10], and to a lesser extent αVβ5 integrin [11], via interaction of the RGD-containing Ad penton base protein. In addition to αVβ3 and αVβ5, other integrin receptors for Ad may include αVβ1, and α5β1 [12]. Because Ad uses both CAR and αV integrins, we used our flow cytometry assay to evaluate CAR expression in platelets and integrin-mediated Ad binding to platelets.

## Results

### Human platelets bind Ad particles

**Figure 1:**
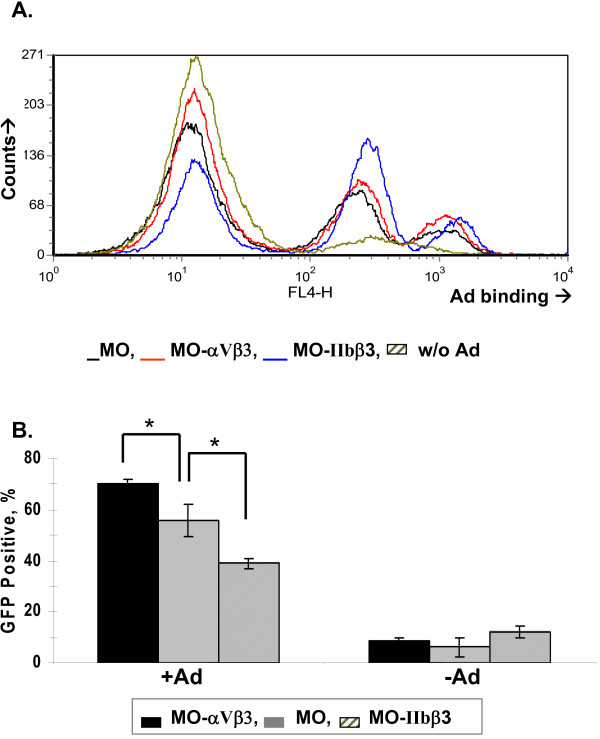
**Flow cytometry to detect Ad attachment to nucleated human cells**. (**a**) One million cells of the isogenic human melanoma cell lines Mo and the stably-transfected Mo-αVβ3 and Mo-αIIbβ3 cell lines (respectively expressing αVβ3 integrin and the platelet αIIbβ3 integrin) were incubated with Ad (MOI = 10, 4°C, 1-hr), followed by rinse and staining with a FITC-labeled anti-Ad hexon antibody. The negative control comprised omitting Ad. Histograms show the distribution and fluorescence intensity of Ad bound to the cell surface (**b**) Ad infection in the above cell lines was studied using a replication deficient Ad vector expressing GFP (AdGFP). Cells were incubated with AdGFP at an MOI of 10 for 4 hours at 37°C, medium replaced and cells further cultured for 18-hrs. Intracellular GFP expression was measured using flow cytometry. *, *p *< 0.05 for enhanced Ad infection of Mo-αVβ3 vs. Mo cells and Mo vs. Mo-αIIbβ3 cell. Representative images of at least 2 different experiments (*n *= 3 for each).

To characterize attachment of Ad group C (serotype 5) to human platelets we employed a direct flow cytometry assay on human platelets using a FITC-labeled anti-Ad hexon antibody (see materials and methods section). First, we calibrated the system measuring Ad attachment to nucleated cells (Fig. 1), derived from isogenic human melanoma cell lines stably expressing either the Ad integrin receptor αVβ3 or the platelet integrin αIIbβ3 [13]. The specific integrin expression profile in these cells was confirmed with indirect flow cytometry (not shown). Ad binding to the cell surface of these cell lines (measured in 4°C) was similar, comprising two main populations, i.e. a small cell population binding Ad with high affinity and a larger population binding Ad with medium affinity (Fig. 1a). Of note, expression of the primary Ad attachment receptor, CAR, was practically absent in Mo cell lines (see below), thereby suggesting that surface integrins suffice to mediate Ad attachment in these cells. To discern in these nucleated cells cell surface Ad binding from infection, we also allowed cell entry (in 37°C) following infection with Ad encoding GFP (AdGFP) and measured transgene expression by direct flow cytometry (Fig. 1b). These distinct flow cytometry assays could clearly differ between αV-enhanced Ad cell entry (Fig. 1b) and αV-independent Ad surface attachment (Fig. 1a).

**Figure 2:**
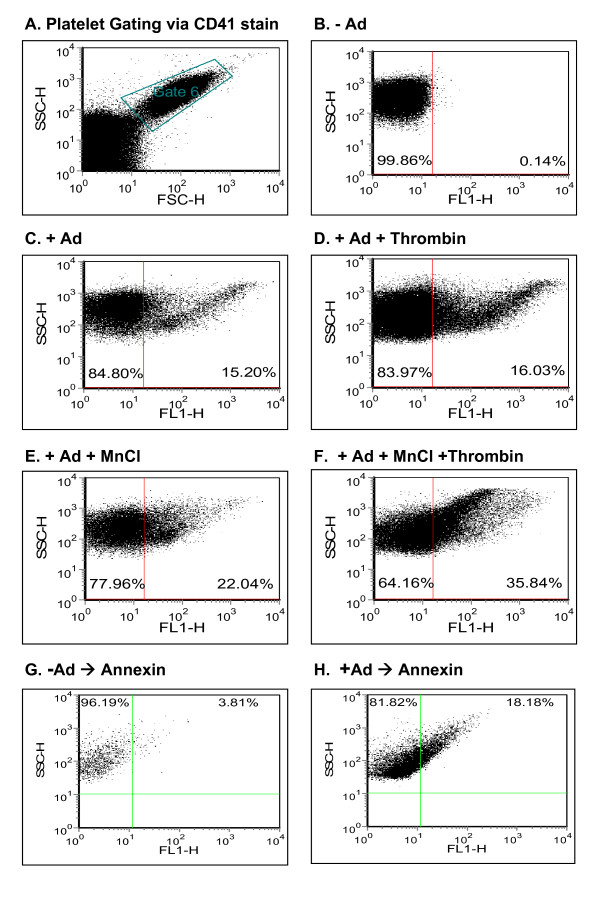
**Characterization of Ad binding to human platelets**. Platelets were isolated from platelet-rich plasma as described in Materials and Methods. Platelets were incubated with Ad (MOI = 10, 1 hr, RT), followed by a thorough rinse and incubation with a FITC-labeled anti-Ad hexon antibody (1:1 dilution, 4°C, 1-hr), Direct flow cytometry was used to measure Ad binding as FITC-positive platelet events (**a**) Platelets were gated by their characteristic forward light scatter and labeling with an anti-αIIbβ3 (α-CD41) antibody. (**b**) To exclude non-specific recognition of unbound platelets by the anti-Ad hexon antibody, the negative control comprised omitting Ad and incubating platelet directly with the antibody. (**c**) The degree of Ad attachment to platelets was measured by staining with the FITC-anti Ad hexon antibody. (**d**) To evaluate the effect of platelet activation on Ad binding, platelets were first activated by thrombin (0.5 U/ml, 20 min, RT), rinsed and incubated sequentially as above with Ad and stained by the anti-Ad hexon antibody. (**e**) To measure the effect of divalent ion supplementation on Ad attachment, MnCl_2 _(5 mM) was added prior to Ad incubation. (**f) **Enhancement of Ad attachment to platelets by sequential thrombin activation and MnCl_2 _supplementation. (**g**, **h**) Ad incubation activates platelets. Platelet activation was measured using annexin staining, reflecting exteriorization of phosphatidylserine, either w/o Ad (**g**) or w/Ad (MOI = 10, RT, 1-hr) (**h**). All figure data representative of at least 2 different experiments (*n *= 3 for each).

Next, we employed direct flow cytometry to detect and characterize attachment of Ad to platelets. To this end, the unique flow cytometry appearance of platelets could allow their specific gating, further confirmed by platelet stain with anti-CD41 (αIIbβ3), an integrin expressed uniquely in platelets (Fig. 2a). Human platelets were incubated with Ad, rinsed and incubated with FITC-labeled anti-Ad hexon antibody prior to flow cytometry. This strategy allowed quantitative identification of Ad particles adherent to the platelet surface (Fig. 2c). There was no cross-reactivity of FITC-labeled anti-Ad hexon antibody with human platelets (Fig. 2b). Platelet activation by thrombin did not affect Ad attachment to platelets (Fig. 2d), and Mn^+2 ^supplementation marginally enhanced Ad attachment (Fig. 2e). However, combining Mn^+2 ^supplementation with thrombin activation substantially enhanced Ad attachment to platelets (Fig. 2f).

**Figure 3:**
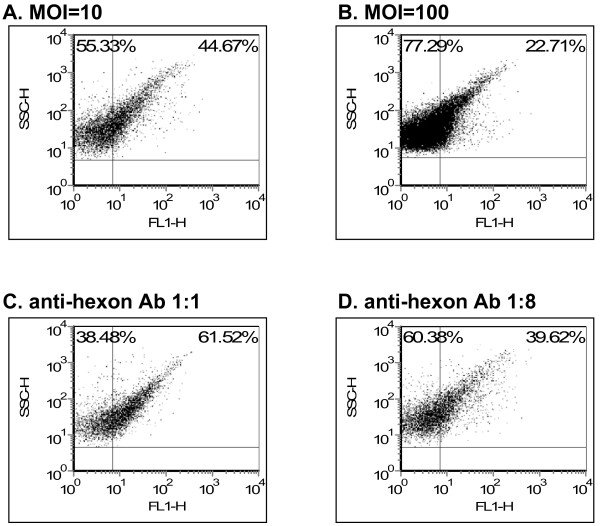
**Optimization of conditions for platelet Ad binding**. Platelets were isolated as above and incubated with Ad (MOI = 10 **(a) **or MOI = 100 **(b)**, 1 hr, RT, 1:1 antibody dilution). Direct flow cytometry was used to measure Ad binding as FITC-positive platelet events. **(c,d) **Optimization of the FITC-labeled anti-Ad hexon antibody dilution (**c**), 1:1 (**d**) 1:8 dilution, RT, 1-hr]. 1:2 and 1:4 antibody dilutions resulted in levels of Ad binding detection between 1:1 and 1:8 (not shown). Figures representative of *n *= 4.

A previous report suggested that *in vitro *incubation of Ad with human platelets failed to aggregate platelets [1]. In contrast, systemic Ad injection could induce platelet activation *in vivo *[2,3] and enhanced platelet clearance [4]. To clarify this issue we measured exteriorization of the platelet membrane phosphatidylserine using annexin stain (indicating apoptosis in nucleated cells but serving as a marker of activation in platelets [14]) and observed that Ad could efficiently activate human platelets *in vitro *(Fig. 2g,h). To optimize the conditions of Ad-platelet binding we tested several MOIs and FITC anti-hexon antibody concentrations (Fig. 3) and found that an MOI of 10 is optimal for Ad binding and that dilution of the FITC anti-hexon antibody resulted in a reduced Ad signal. Thus, Ad attachment to human platelets can be characterized *in vitro *using direct flow cytometry.

### Ad virions adhere to the platelet surface

**Figure 4:**
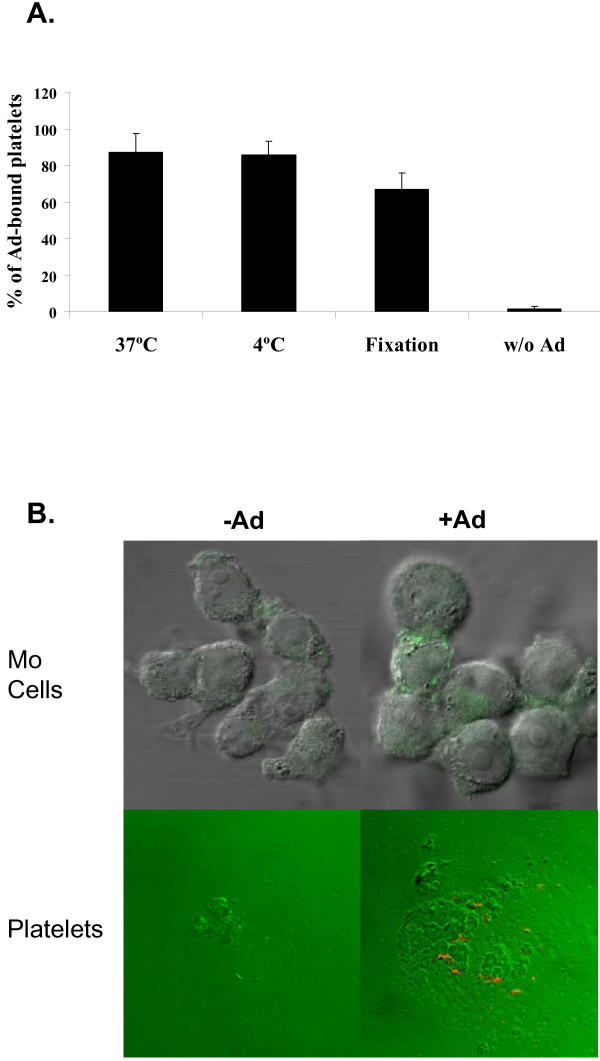
**Ad binds to the platelet cell surface**. **(a) **Platelets were incubated with Ad (MOI = 10, 2-hrs) at 4°C or 37°C to compare cell surface binding (4°C) vs. potential cell entry (37°C). Alternatively, platelets were fixed in 4% paraformaldehyde and then measured for cell surface Ad binding using direct flow cytometry. (**b**) To qualitatively evaluate cell surface Ad binding, Mo cells and platelets were incubated with Ad (MOI = 10, 4°C for Mo cells and RT for platelets, 1-hr) or mock-infected, rinsed, mounted on a cover slip, fixed, blocked with BSA, rinsed, incubated with the FITC-anti-hexon antibody and visualized with a confocal fluorescent microscope. Ad virions adherent to the cell surface were detected as green labeling in Mo cells and orange labeling in platelets. Negative controls included omission of Ad incubation.

To discern between platelet cell entry vs. Ad attachment to the platelet membrane, we employed two methods. First, Ad was incubated with platelets either at 37°C or 4°C, the latter precluding cell entry [10]. In addition, Ad were incubated either with live or fixed platelets, the latter also precluding cell entry. Our data indicate that Ad-platelet interaction solely involves adherence to the cell surface (Fig. 4a). Confocal immunofluorescent microscopy qualitatively confirmed attachment of Ad virions to the platelet surface (Fig. 4b).

### Ad binds to platelet surface integrins

**Figure 5:**
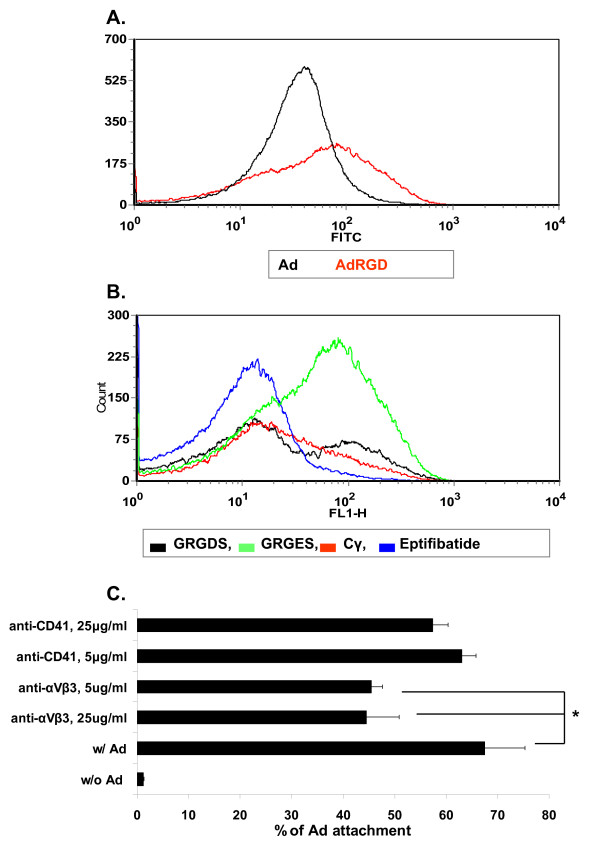
**Ad binds to human platelets integrin-dependently**. **(a) **Platelets were isolated, incubated with Ad (black) or AdRGD (red) (MOI = 10, RT) and stained with FITC-labeled anti-hexon antibody as above in Fig. 2. **(b) **Prior to incubation with AdRGD, platelet integrins were blocked (RT, 1-hr, 150 mg/ml) with the peptides GRGDS (RGD), eptifibatide (a synthetic RGD analog) or Cγ (a 12-amino acids peptide derived from the carboxy terminus of the FBG γ chain). GRGES served as a negative control. **(c) **Platelets were first incubated with monoclonal anti-αvβ3 or anti-CD41 (=αIIbβ3) antibodies (at 5 or 25 mg/ml), prior to rinse and incubation with AdRGD, rinse and staining with anti-hexon antibody. *, *p *< 0.05 for inhibition of Ad attachment with anti-αvβ3 antibody. Representative images of at least 2 different experiments (*n *= 3 for each).

Next, we employed flow cytometry to evaluate the mechanism of Ad binding to the platelet surface. Ad displaying an RGD ligand in the HI fiber knob loop adhered more efficiently to platelets than unmodified Ad (Fig. 5a) and a variety of RGD-based ligands could block Ad attachment to platelets (Fig. 5b). However, GRGDS (RGD) was less efficient vs. eptifibatide (a synthetic analog based on the barbourin motif, containing a homoArginine-Glycine-Aspartate sequence), or vs. the FBG carboxy terminus 400–411 dodecapetide (Cγ). These two peptides could efficiently (6-fold) block platelet Ad binding. A monoclonal anti-αVβ3 antibody could also specifically, but only partially, block Ad attachment to platelets (Fig. 5c). Because αVβ3 expression on platelets is minute [15], these data may indicate a high affinity of Ad to platelet αVβ3. Of note, while previous studies showed a role for heparan sulfate proteoglycans in Ad binding to nucleated cells [12], heparan sulfate does not appear to play a role in Ad binding to platelets as several doses of heparin did not block Ad attachment to platelets (not shown).

**Figure 6:**
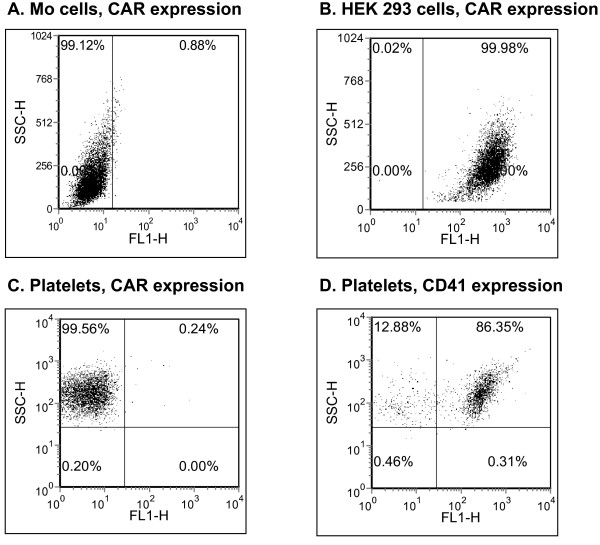
**CAR expression in platelets**. CAR expression was measured using indirect flow cytometry with a polyclonal rabbit anti-CAR antibody and a secondary FITC labeled antibody. HEK293 cells and Mo melanoma cells served as positive and negative controls for CAR expression, respectively. αIIbβ3 (CD41) expression in normal platelets served as a positive control for platelet receptor expression.

Expression of CAR, the primary Ad attachment receptor on nucleated cells, has been recently reported in platelets [2]. However, in our studies CAR expression was not detected on human platelets (Fig. 6), confirmed by negative (Mo cells) and positive (HEK 293 cells) nucleated cell controls for CAR expression. Taken together, our data indicate a role for integrins in mediating Ad binding to human platelets *in vitro*.

### Enhanced Ad attachment in Glanzmann Thrombasthenia platelets

**Figure 7:**
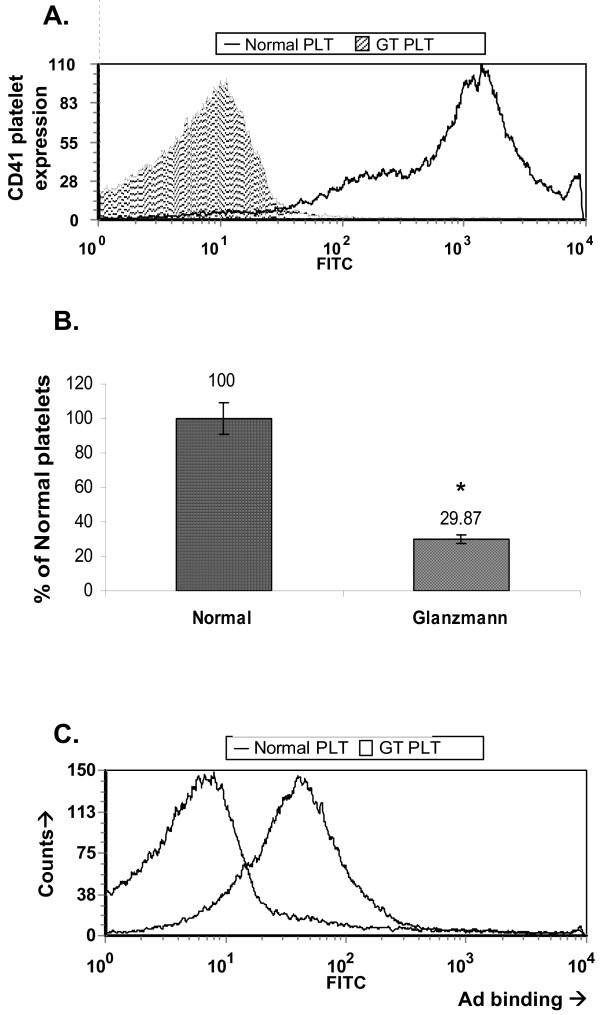
**Glanzmann thrombasthenia platelets efficiently bind Ad**. (**a**) Platelets from a patient with Glanzmann thrombasthenia (GT PLT) and normal platelets (Normal PLT) were analyzed for αIIbβ3 expression using indirect flow cytometry. (**b**) Functional αIIbβ3 deficiency of GT platelets was confirmed by a fibrinogen (FBG) attachment assay. (**c**) Ad attachment was measured in normal human platelets vs. GT platelets by direct flow cytometry as above in Fig. 2 except for an MOI of 5. *, *p *< 0.05 for impaired platelet attachment to FBG. Representative images of at least 2 different experiments (*n *= 3 for each).

We next sought to evaluate Ad binding to platelets deficient of the integrin αIIbβ3 (also called gpIIb/IIIa) from a patient with Glanzmann thrombasthenia (GT). GT mutations in the αIIbβ3 gene result in a bleeding tendency because αIIbβ3 expression is either abolished or the fibrinogen (FBG) binding domain is disrupted [1]. Consequently, platelet adhesion and aggregation are impaired, resulting in life-long bleeding diathesis. The mutation in the kindred to which this patient belongs comprises complete abolition of αIIβ expression while α3 expression is maintained to a small extent. Thus, a small degree of αVβ3 platelet expression may be preserved in GT [15]. We first confirmed that platelets from the patient with GT had practically no αIIbβ3 expression (Fig. 7a), and were functionally impaired as evident by both reduced platelet attachment to FBG (Fig. 7b) and decreased activation by thrombin (not shown). Surprisingly, GT platelets bound Ad 5-fold more efficiently than normal platelets (Fig. 7c).

## Discussion

Platelets bind physiological ligands in an RGD-dependent manner, e.g. FBG, von Willerband factor (VWF), fibronectin and vitronectin. Non-physiological platelet integrin ligands include disintegrins (cyclic RGD-based polypeptides in snake venoms) and a number of microorganisms. One of the most extensively characterized pathogens with respect to nucleated cellular integrin interaction is Ad. Cell entry by Ad viruses initially involves attachment of the Ad fiber knob to the primary Ad receptor, CAR [6], followed simultaneously or subsequently by binding of any of the five RGD protrusions on the Ad penton base protein to cellular αV integrins heterodimerized to specific β chains [10,11]. A critical requirement for Ad infection is the interaction of membrane αV integrin with the RGD-displaying Ad penton base. This interaction has been previously demonstrated via inhibition of Ad cell entry by RGD peptides and antibodies to αV integrins [10,16]. Integrin receptors are heterodimers comprised of α and β subunits whose specific sequence and activation-dependent conformation determine their ligand affinity. The ligand motif for a number of integrins is based on an arginine-glycine-aspartate (RGD) sequence and variations on the RGD theme determine specific ligand-integrin recognition. For example, fibrinogen (FBG) binding to αIIbβ3 depends on prior inside-out signaling, resulting in platelet priming and conformational αIIbβ3 transition into a high-affinity state [17].

In the current study, we developed a direct flow cytometry approach to characterize Ad binding to human platelets, focusing on platelet integrin-mediated binding. Optimization of the methodology could show a number of pertinent findings. First, Ad binding to human platelets can be manipulated *in vitro *by combining a divalent ion and thrombin activation (Fig. 2). Second, Ad binding activates platelets *in vitro *(Fig. 2). Third, an optimal MOI in the order of 10 (Fig. 3) was observed for Ad attachment to the platelet surface (Fig. 4). This optimal MOI is compatible with the ratio of 40 between the spherical surface areas of platelets and Ad, given respective diameters of ~3 m and ~150 nm. Fourth, Ad attachment to human platelets is at least partially mediated by platelet integrins, as evident by blocking assays using anti-αVβ3 monoclonal antibody and RGD peptidomimetics (Fig. 5).

Fifth, although CAR was previously reported to be expressed in human platelets both at the level of RNA and using flow cytomtery [2], our studies show CAR deficiency in normal human platelets (Fig. 6).

Because CAR mediates homotypic cell adhesion, it is generally present in specialized intracellular junctions, including the cardiac intercalated disk and the adherens junction of polarized epithelial cells [12]. Although CAR is abundantly expressed in epithelial cells during embryogenesis, its expression in adult mice is restricted to fewer cell types, contrasting with the homogeneous expression pattern of αV-integrins [18]. Thus, in bone marrow hematopoeitic lineages CAR expression is minute [19,20]. Othman et al employed the RmcB anti-CAR antibody and did not report a CAR-negative cell line to demonstrate the specificity of the anti-CAR antibody [2]. In contrast, we confirmed specificity of the rabbit H-300 polyclonal anti-CAR antibody in both CAR-positive and negative cell lines prior to testing CAR expression in platelets. While variations in the specificity of the anti-CAR antibodies employed may account for the discrepancy between our results and Othman et al [2], further studies are required to conclusively define CAR expression in human platelets. However, our blocking assays, along with the recent observation that Ad serotype 11 can efficiently bind to mouse platelets fiber-independently [21], further highlight the role of platelet integrins as mediators of Ad binding. Other integrins expressed by platelets include a5b1 and a1b1 [22,23]. While these are not well established as Ad receptors, the recent finding of Ad interference with platelet adhesion to fibronectin [2] may suggest that Ad may also bind to the fibronectin receptor a5b1.

Previously, αIIbβ3 (gpIIb/IIIa), the primary platelet FBG receptor was reported to mediate platelet attachment of the intracellular bacterial microorganisms, chlamydia and borrelia. These studies employed blocking assays using abciximab (an anti- αIIbβ3 antibody) and RGD peptides [24-26], at 4–400 fold higher blocking concentrations than employed in this study. However, while αVβ3 may partially mediate attachment of Ad to platelets (Fig. 5), αIIbβ3 does not appear to play a significant role in Ad binding to platelets, as evident by lack of blockade by a monoclonal antibody against αIIbβ3 (Fig. 5), and by avid adherence of Ad to αIIbβ3-deficient platelets from a patient with Glanzmann thrombasthenia (Fig. 7). Of note, unlike borrelia binding to platelets that requires prior platelet activation [24,25], Ad could also efficiently bind to naïve platelets, although platelet activation along with MnCl enhanced Ad binding (Fig. 2).

Glanzmann thrombasthenia (GT) is a rare, inherited disorder of platelet function characterized by mucocutaneous hemorrhage caused by mutations in the αIIbβ3 gene. The major laboratory finding in GT is a profound defect in platelet aggregation caused by a failure of αIIbβ3 to bind FBG. In this study we observed minute expression of αIIbβ3 on platelets from a patient with GT. A typical mutation in this patient's kindred was previously found to completely abolish αIIβ expression while a very low β3 (IIIα) expression level was still detected [15]. Thus, unlike other αIIbβ3 mutations in other GT kindreds where both αIIβ and β3 were completely absent, platelets from this GT subpopulation maintain the potential to express some αVβ3 [15]. In this context, while we failed to document substantial αVβ3 expression on normal platelets, Coller et al had measured ~100 αVβ3 receptors per platelet, i.e. 0.25% of the number of αIIbβ3 receptors per platelet [15].

Our findings on platelet-Ad interaction *in vitro *may have implications on the biodistribution of Ad *in vivo*. Previously, partial platelet depletion did not alter Ad biodistribution and it was postulated that Ad attachment to platelets may occur only in a small fraction of platelets [24]. However, we speculate that blockade of platelet integrins *in vivo *will alter Ad biodistribution. Recently, attachment of Ad particles to platelets resulted in platelet-leukocyte aggregates [3], VWF and p-selectin-mediated thrombocytopenia [2] via clearance by the reticuloendothelial system and the complement pathway [27]. While this scenario may complicate Ad-based gene delivery, it may reflect an evolutionary-conserved defense mechanism allowing to efficiently clear circulating RGD-displaying microorganisms such as Ad. In support of this hypothesis, RGD display on the Ad fiber knob, in addition to the natural RGD ligand on the Ad penton base, has been reported to result in paradoxically diminished systemic tissue distribution [16]. Thus, fundamental to future rationalized systemic Ad-based gene delivery endeavors in humans is the molecular characterization of Ad-platelet interaction *in vivo*. Additionally, Ad biodistribution and toxicity may differ in GT patients from healthy subjects.

Taken together, we report a direct flow cytometry assay to characterize Ad binding to platelets. This approach may eventually be employed to determine the exact integrin profile accounting for Ad attachment to human platelets and therefore may have implications on systemic Ad biodistribution.

## Methods

### Adenoviral vectors

Ad vectors used in the attachment studies were E1/E3 deleted, replication-deficient serotype 5 Ad vectors. Ad-RGD vector is a caspid-modified Ad, displaying a CDCRGDCDC ligand in the Ad capsid fiber (both from David T. Curiel, University of Alabama at Birmingham). Vector titer was determined by both plaque forming units (PFU) and by spectrophotometric measurement of DNA optical density at 260 nm. The PFU/viral particle ratio was ~100 for the Ad vectors.

### Attachment assays

All attachment studies were performed in suspension after initial blocking with BSA. Antibody and peptide blocking assays were performed at 4°C (room temperature [RT] for platelets) with pre-incubation for 1-hr at 5 and 25 μg/ml or 150 μg/ml, respectively, as previously described to block cellular αV integrins [10,11]. Next, cells were rinsed, incubated with Ad for 1-hr, rinsed and processed for flow cytometry after incubation on ice with FITC-labeled anti-Ad hexon antibody for 1-hr. Attachment of Ad particles to the various Mo cell lines was measured after detaching cells by minimal trypsinization, rinse, resuspension (1 × 10^6^) in serum-free medium, incubation with Ad (MOI = 10) at 4°C for 1-hr, rinse and incubation with FITC anti-hexon antibody for 1-hr on ice followed by processing for flow cytometry. Specificity of Ad attachment was confirmed via omission of Ad.

### Antibodies and integrin inhibitors

Anti CD41 (clone 5B12) is a goat monoclonal anti-gpIIb/IIIa antibody (Dako, Carpinteria, CA). LM609 is a function-blocking anti- αVβ3 antibody (Chemicon, Temecula, CA). FITC-labeled anti-Ad hexon antibody was from Chemicon. A rabbit polyclonal anti-CAR antibody (H-300) was from Santa Cruz biotechnology (Santa Cruz, CA). FITC-labeled secondary antibodies were from Jackson Immunoresearch laboratories. FBG was purchased from Sigma. Eptifibatide (Integrilin^®^, COR therapeutics Inc, South San Francisco, CA) is a small FBG mimetic primarily antagonizing αIIbβ3 (gpIIb/IIIa), but also αV integrins [28]. Other FBG peptidomimetic blockers were GRGDS and the FBG-specific 12 amino acid carboxy terminus of the human FBG gamma chain (Cγ), HHLGGAKQAGDV, and the control GRGES peptide, all synthesized by Biosight (Carmiel, Israel).

### Cells

The isogenic cell lines Mo, Mo-αVβ3 and Mo-αIIbβ3 were a kind gift from Mark H. Ginsberg (The Scripps Research Institute, La Jolla, CA) [13]. These cell lines include the αV-deficient parental Mo melanoma cell line originally derived from the human melanoma M21 cell line. Mo cells express β3 integrin mRNA and protein but neither αV or αIIβ. Mo- αVβ3 and Mo-αIIbβ3 were generated by Dr. Ginsberg and colleagues from Mo cells by stable expression of αV or αIIβ, respectively [13]. Thus, while Mo cells express neither αVβ3 nor αIIbβ3, Mo-αVβ3 and Mo-αIIbβ3 (=Mo αIIβ) express αVβ3 and αIIbβ3, respectively [29]. We confirmed the specific integrin expression for each cell line (not shown). Of note, the platelet integrin αIIbβ3 is also known as gpIIb/IIIa or CD41.

### Platelet processing

Human blood was collected from healthy, medication-free donors in tri-sodium citrate 3.8%, 1:7 ratio. Platelet rich plasma (PRP) was prepared as before [30] with the following modifications. Briefly, blood was centrifuged at 800 rpm for 15 minutes, PRP collected and further centrifuged at 5000 rpm for 4 minutes in the presence of citrate (5 mM). The platelet pellet was resuspended in magnesium-free and calcium-free PBS with citrate (final concentration 5 mM). Platelets were counted and studied microscopically to exclude aggregates and contaminating cells. When indicated, thrombin (Sigma, 0.5 U/ml, 20 min, RT) was used to activate platelets. Platelet activation was measured by annexin v flow cytometry [14]. To evaluate Ad attachment to gpIIb/IIIa-deficient platelets, following informed consent, blood was obtained from a previously-diagnosed, 31-yr-old male with Glanzmann thrombasthenia (GT), simultaneously with blood drawn from a healthy control and processed as above. GT platelets manifested deficient gpIIb/IIIa expression, platelet attachment to FBG and activation. FBG-platelet attachment assay was performed in 96-well plates after pre-coating with 50 ml of 50 microgram/ml FBG solution overnight at 4°C and blocking with BSA 1%. Normal or GT platelets (2 × 10^6^) were then incubated for 1-hr at 37°C, rinsed, fixed and counted.

### Flow cytometry

Indirect flow cytometry was performed to measure membrane-bound Ad particles. Platelets were pre-defined and gated by both their characteristic forward light scatter and labeling with an anti-CD41 (αIIbβ3) antibody. Binding of Ad particles to platelets was identified by FITC fluorescence above a threshold value set as the maximal background fluorescence, predetermined by analysis of platelet FITC fluorescence, as previously described for other microorganisms [24-26].

Ad binding to nucleated cells was evaluated similarly with the exception that cells were diluted to 1 × 10^6^/ml and Ad incubation was performed at 4°C for 1-hr [10]. Event data were collected from each sample with scatter data in linear mode and fluorescent data in logarithmic mode. Data were analyzed using forward and side scatter gates to exclude dead cells and cell fragments. Fluorescence histograms or dot plots were generated from the gated population and percentage of positive events was used to determine differences between cell samples.

### Immunofluorescence

Cells and normal platelets were incubated with Ad (MOI = 10, 4°C, 1-hr) or mock-incubated, mounted on a cover slip and visualized with a confocal fluorescent microscope. FITC-positive labeling was observed only in cells incubated with Ad.

### Statistical analysis

Experiments were repeated two to three times. Representative flow cytometry images are presented. Where indicated, *p *< 0.05 was considered statistically significant.

## Abbreviations

gpIIb/IIIa: αIIbβ3; Ad: Adenovirus; FBG: fibrinogen; Cγ: carboxy terminus of the human FBG gamma chain; CAR: Coxsackie adenoviral receptor; GT: Glanzmann thrombasthenia; MOI: multiplicity of infection; RT: room temperature.

## Competing interests

The authors declare that they have no competing interests.

## Authors' contributions

NS designed study, performed experiments and wrote manuscript, GE performed experiments, DKG intellectual contribution and reagents, LK performed experiments, SUS intellectual contribution and provided cell lines, YSH designed experiments and wrote manuscript.
